# *Burkholderia* vaccines: are we moving forward?

**DOI:** 10.3389/fcimb.2013.00005

**Published:** 2013-02-05

**Authors:** Leang-Chung Choh, Guang-Han Ong, Kumutha M. Vellasamy, Kaveena Kalaiselvam, Wen-Tyng Kang, Anis R. Al-Maleki, Vanitha Mariappan, Jamuna Vadivelu

**Affiliations:** Department of Medical Microbiology, Faculty of Medicine, University of MalayaKuala Lumpur, Malaysia

**Keywords:** *Burkholderia pseudomallei*, *Burkholderia mallei*, *Burkholderia cepacia*, melioidosis, glanders, cystic fibrosis, vaccine

## Abstract

The genus *Burkholderia* consists of diverse species which includes both “friends” and “foes.” Some of the “friendly” *Burkholderia* spp. are extensively used in the biotechnological and agricultural industry for bioremediation and biocontrol. However, several members of the genus including *B. pseudomallei, B. mallei*, and *B. cepacia*, are known to cause fatal disease in both humans and animals. *B. pseudomallei* and *B. mallei* are the causative agents of melioidosis and glanders, respectively, while *B. cepacia* infection is lethal to cystic fibrosis (CF) patients. Due to the high rate of infectivity and intrinsic resistance to many commonly used antibiotics, together with high mortality rate, *B. mallei* and *B. pseudomallei* are considered to be potential biological warfare agents. Treatments of the infections caused by these bacteria are often unsuccessful with frequent relapse of the infection. Thus, we are at a crucial stage of the need for *Burkholderia* vaccines. Although the search for a prophylactic therapy candidate continues, to date development of vaccines has not advanced beyond research to human clinical trials. In this article, we review the current research on development of safe vaccines with high efficacy against *B. pseudomallei, B. mallei*, and *B. cepacia*. It can be concluded that further research will enable elucidation of the potential benefits and risks of *Burkholderia* vaccines.

## *Burkholderia* spp.

The genus *Burkholderia* is a phylogenetically coherent genus consisting of interesting and complex bacterial taxonomy which includes a wide variety of Gram-negative, bacilli (1–5 μm length and 0.5–1.0 μm width), that are motile due to the presence of multi-trichous polar flagella (Vandamme et al., 2007). These bacteria are generally obligate aerobes and commonly found in the soils of all temperatures including the Arctic soil at temperature of 7°C (Master and Mohn, 1998) and in groundwater worldwide (Ussery et al., 2009). The genomic G + C content of *Burkholderia* spp. *ranges* between 64% and 68.3% (Yabuuchi et al., 1992). Members of the genus *Burkholderia* were formerly classified as belonging to the genus of *Pseudomonas* which belongs to the Proteobacteria homology group II (Yabuuchi et al., 1992). The taxonomy of the genus *Burkholderia* has undergone considerable changes since it was first reported, when 22 validly described species were included (Coenye et al., 2001). Currently, the *Burkholderia* genus comprises at least 43 species, which are extremely diverse and versatile (Vial et al., 2007; Compant et al., 2008).

Members of the genus *Burkholderia* can form associations with plants and are also able to cause disease in animals and humans. There are two main factors that can be attributed to the ecological versatility of the members of this genus which includes: (1) the huge coding capacity of their large multireplicon genomes (6–9 Mb) that allow the members of the genus to be metabolically robust; and (2) an array of insertion sequences in their genomes which promote genomic plasticity and general adaptability (Lessie et al., 1996). Their survival and persistence, in the environment and in host cells, offers a notable example of bacterial adaptation (Woods and Sokol, 2006).

Several members of the genus are used in a variety of biotechnological applications, including bioremediation, biological control of plant diseases, water management, and also improvement of nitrogen fixation (Parke and Gurian-Sherman, 2001). Although most of the species in the genus *Burkholderia* are not pathogenic for healthy individuals, a few that include *B. pseudomallei, B. mallei*, and *B. cepacia*, are capable of causing severe, life-threatening infections in both normal and immunocompromised individuals. Some of these infections are inherently difficult to treat due to the resistance of these bacteria to multiple antibiotics, the ability to form biofilms, and the establishment of intracellular and chronic infection stages in the host (George et al., 2009).

Preventive measures such as the use of vaccines for active immunization could offer significant protection to persons living in endemic areas and reduce the worldwide incidence of *Burkholderia* infections. In addition, the range of infections caused by *Burkholderia* spp. and the potential to develop chronic infections in the immunocompromised host through the persistent nature of these bacteria makes the need for a vaccine crucial. In recent years, many studies have been performed in order to identify vaccine strategies against *B. pseudomallei, B. mallei*, and *B. cepacia*, but to date no ideal candidate has yet emerged (Sarkar-Tyson and Titball, 2010). The most important reason for the failure to identify a good vaccine is due in the main to the intracellular nature of these pathogens which makes it difficult to evoke both an antibody response and also strong cellular-based responses.

### Burkholderia pseudomallei

*B. pseudomallei* is endemic in the soils of southeast Asia and northern Australia and have been reported to occur in other tropical and subtropical regions, worldwide. The bacterium is now classified as Category B priority agent and the disease caused is known as melioidosis which was first described as a “glanders-like” disease among morphine addicts by Whitmore and Krishnasawami in Rangoon, Burma in 1911 (Whitmore, 1913; Whitmore and Krishnaswami, 2012).

Melioidosis presents as a broad range of conditions from acute fulminant pneumonia and septicemia acquired by inhalation to wound infections acquired through inoculation of the bacteria from soil through skin abrasion (Currie et al., 2000; Dance, 2002). *B. pseudomallei* also poses a worldwide emerging infectious disease problem and a bioterrorism threat due to its severe course of infection, aerosol infectivity, low infectious dose, an intrinsic resistance to commonly used antibiotics, lack of a currently available vaccine, and the worldwide availability (Stevens et al., 2004).

Pathogenesis of the disease has been demonstrated to include the ability of *B. pseudomallei* to escape into the cytoplasm from endocytic vacuoles of the host cells, where it can polymerize actin and subsequently spread from one cell to another (Kespichayawattana et al., 2000; Breitbach et al., 2003; Stevens et al., 2005; Boddey et al., 2007). A major feature of this bacterium is the ability to remain latent in the host and cause recrudescent or relapsing infections following many years past the initial infection. This relapse of the infection is quite common despite appropriate antibiotic therapy and the presence of high antibody titers in infected patients (Currie et al., 2000; Vasu et al., 2003). Regardless of antibiotic therapy, rapid progress of acute melioidosis to sepsis, followed by death within 48 h of clinical onset has been reported (Cheng et al., 2007). Mortality rate of patients with septic shock is reported to be approximately 80–95% (Leelarasamee, 2004).

### Burkholderia thailandensis

*B. thailandensis* is a closely related yet distinct *B. pseudomallei*-like organism (Brett et al., 1998). Although there is 99% similarity of *B. thailandensis* genes with its virulent counterpart, the pathogenic species *B. pseudomallei*, it is apparently non-pathogenic in humans. Smith et al. (1997) have reported that *B. thailandensis* demonstrated a mean 50% lethal dose of 10^9^ cfu/mouse compared to 182 cfu/mouse for *B. pseudomallei*, indicating that it is also much less virulent in animal models. The avirulent properties of *B. thailandensis* might be attributed to the presence of a complete arabinose biosynthesis operon, which is an anti-virulence property and this is largely deleted in the *B. pseudomallei* genome (Moore et al., 2004; Lazar Adler et al., 2009). However, like *B. pseudomallei, B. thailandensis* also has an intracellular lifecycle. Thus, it is often used as a model microbe to study various aspects of the potential bioterrorism agent *B. pseudomallei* and *B. mallei*.

### Burkholderia mallei

Glanders is caused by *B. mallei* which typically infects solipeds, such as horses, mules, and donkeys. *B. mallei* only occasionally infect humans such as laboratory workers and personnel who are often in close contact with infected animals (Srinivasan et al., 2001). Due to its ability to infect via inhalation route, the bacterium was among the first bioweapon used during both World Wars I and II. Since then, it has been classified as a Category B priority agent (Wheelis, 1998).

The course of infection is reliant on the route of exposure including ingestion of contaminated poultry, inhalation of aerosol containing the bacterium and abrasion which leads to a localized cutaneous infection. In an acute infection, general symptoms include fever, malaise, abscess formation, pneumonia, and sepsis. In cases of untreated septicemic infections, the fatality rate is as high as 95% whereas for antibiotic-treated individuals, 50% fatality has been reported (Currie, 2009). Despite numerous reports that indicate *in vitro* susceptibility of *B. mallei* to a wide array of antibiotics, the *in vivo* efficacies are not well-established (Kenny et al., 1999; Heine et al., 2001; Judy et al., 2009). Although the pathogenic determinants of *B. mallei* have already been defined, the pathogenesis of the bacteria is still unknown.

### Burkholderia cepacia

*B. cepacia* was first described as a phytopathogen with the ability to infect onion bulbs causing a condition called “soft rot” (Burkholder, 1950). The *B. cepacia* complex (Bcc) is a collection of closely related species which are genotypically distinct but phenotypically similar species. Strains originally identified as *B. cepacia* were classified into at least nine genomovars, which are referred to as the Bcc (Vandamme et al., 1997, 2000) and more recently, another eight genomovars have been included (Vanlaere et al., 2008, 2009). All Bcc species share a high degree of 16S rDNA sequence similarity (98–99%) and moderate levels of whole genome DNA–DNA hybridization (30–50%) (Vandamme et al., 1997, 2000; Vanlaere et al., 2009).

*B. cepacia* is naturally found in moist soil, particularly in the rhizosphere of plant and freshwater environments in relatively high population. It is now recognized as a significant human pathogen associated with nosocomial infections, especially among cystic fibrosis (CF) patients (Cepacia syndrome) or chronic granulomatous disease (CGD) which can lead to rapid decline of lung function. Even with proper treatment the Cepacia syndrome has been known to lead to death within a few weeks of infection. CF patients might be asymptomatic carriers of *B. cepacia* which over time can cause the infection to progress into a necrotizing pneumonia and bacteremia. In CF patients, *B. cepacia* infections lead on to multi-organ system dysfunction, the most severely affected being the lower respiratory tract of the affected patients. Person-to-person transmission from one CF patient to another via close contact in a nosocomial environment has been reported (Govan et al., 1993; Jones et al., 2004). There are also other reports suggesting the involvement of *B. cepacia* infections in cancer and HIV patients as well as among immunocompetent individuals (Marioni et al., 2006; Mann et al., 2010). *B. cepacia* strains have also been isolated in some cases of chronic suppurative otitis media, pharyngeal, and neck infections from immonocompromised individuals as reported by Marioni et al. (2006).

## The need for *Burkholderia* vaccines

Vaccines against *Burkholderia* infections are necessary to offer prophylactic protection for susceptible large populations living in endemic areas worldwide and also for visitors to these areas (Sun et al., 2005; Suparak et al., 2005). Through vaccination, the incidence of persistent or chronic infections in the population can be reduced. As these bacteria occur in the soil, every individual has the potential risk to acquire these infections through routine daily activities.

Compounding this fact, the treatment of *Burkholderia* infections are especially difficult due to the inherent resistance of *Burkholderia* spp. to multiple antibiotics including aminoglycosides, quinolones, polymyxins, and β-lactams (Aaron et al., 2000; Martin and Mohr, 2000; Leitao et al., 2008; Estes et al., 2010). The multi-resistance property of the *Burkholderia* spp. may result from various efflux pumps that efficiently remove antibiotics from the cell, decreased contact of antibiotics with the bacterial cell surface due to their ability to form biofilms, and changes in the cell envelope that reduce the permeability of the membrane to the antibiotic (George et al., 2006).

Therefore, the best way to protect individuals at high risk is through the development of efficacious vaccines which are able to evoke sterilizing immunity or delay the onset of bacteremia and septic shock, to extend the window of opportunity for antibiotic treatment and still be of significant clinical benefit. To date, there is very little information available on the seroepidemiology of melioidosis in the endemic community (Finkelstein et al., 2000; Wuthiekanun et al., 2008). A seroepidemiological study performed in Khon Khean, Thailand, demonstrated that although a majority of the children were seropositive toward *B. pseudomallei*, none of them expressed symptoms of melioidosis, indicating the latency of *B. pseudomallei* in human host (Kanaphun et al., 1993). Additionally, a study conducted in Malaysia revealed seropositivity among melioidosis patients and healthy individuals (Vadivelu et al., 1995). With this existing herd immunity in endemic areas, the development of vaccines would be beneficial.

## Hurdles in development of *Burkholderia* vaccines

At present, no efficacious vaccines are available to prevent infections caused by *Burkholderia* spp. as none are able to achieve sterilizing immunity (Elkins et al., 2004). Despite reports on the ability of several *Burkholderia* vaccines to confer some immunoprotection, none of the vaccines have reached the clinical trial state indicating that there are, major difficulties that exist in the development of safe vaccines against *Burkholderia* infections (Elkins et al., 2004).

An important strategy to consider is the ability of the host to generate a specific immune response in the respiratory tract essentially so that it could prevent the early steps of colonization and infection by these bacteria, thus preventing or reducing lung damage due to inflammation during subsequent infections. Another dilemma in this scenario is, unlike infectious model agents, like *Escherichia coli* or *Salmonella typhimurium*, the pathogenesis of *Burkholderia* spp. and the specific mechanisms by which these bacteria subvert the host defense mechanisms are not well understood. Clinical manifestations of melioidosis are also varied widely from asymptomatic seroconversion, acute manifestation of septicemia, localized abscesses, disseminated infections which lead to concomitant pneumonia and multiple organ abscesses to quiescent latent infection. However, to date, the mechanisms and involvement of altered metabolism in latency is still unclear. Whether the clinical manifestations of the patient were determined by the host, pathogen, or the outcome of the interactions of both parties needs to be clarified (Gan, 2005).

To further compound this issue, *Burkholderia* spp. are facultative intracellular pathogens that are able to invade non-phagocytic cells which leads to the evasion of the humoral immune responses (Lazar Adler et al., 2009). Therefore, elimination of intracellular *Burkholderia* spp. is highly reliant on the cell-mediated immune responses. In addition, clearance of *B. pseudomallei* in patients has also been shown to be ineffective despite the presence of immunoglobulin G_1_ (IgG_1_) response, suggesting the stimulation of Th2 immune response (Puthucheary, 2009). This complicated host–pathogen interaction confers major challenges in the rational design of effective and safe vaccines for use.

Vaccine trial studies in animals have demonstrated development of immunity against *Burkholderia* spp. in the vaccinated animals. Despite developing immunity in animal vaccine studies, postmortem results have indicated the presence of bacteria in multi organ systems of the vaccinated animals indicating that the sterilizing immunity which is the ultimate goal of vaccine was unable to be achieved in most cases. Another proviso to these studies was the finding that experimental vaccine candidates available were only efficient in protecting against acute *Burkholderia* infections but not against chronic *Burkholderia* infections (Patel et al., 2011).

With these issues in the background and the lack of knowledge on the host–pathogen interactions being the most prominent hindrance in the development of vaccine against *Burkholderia* spp., to date no effective vaccines have been made available.

## Animal models for *Burkholderia* vaccines development

In order to study the efficacy of any vaccine, a suitable infection model is a pre-requisite. To date, although different animal models have been used for *Burkholderia* spp. infection studies, among the most extensively developed and described models available are those used for studies on *B. pseudomallei*. The models widely used for *B. pseudomallei* experimentation include the rodent models, especially the inbred mice strains, BALB/c and C57BL/6, and the Syrian golden hamster (Brett et al., 1997; Fritz et al., 1999; Warawa and Woods, 2005).

The use of Syrian golden hamster in *Burkholderia* infection and vaccines development studies are limited since it is not as well characterized as the BALB/c and C57BL/6 model. The Syrian golden hamster model is also highly susceptible to *Burkholderia* infection and unable to mimic human infection (Bondi and Goldberg, 2008).

In melioidosis, it has been demonstrated clinically that release of proinflammatory cytokines in human leads to the pathological changes in acute melioidosis patients. The levels of proinflammatory cytokines also correlated with severity of acute melioidosis in patients (Wiersinga and Van Der Poll, 2009). Similarly, in BALB/c mice, a surge of proinflammatory cytokines levels peaking at 24–48 h after infection was observed. In addition, there was also poor recruitment of lymphocytes and macrophages to the infection site leading to inefficient clearance of *B. pseudomallei* resulting in a failure to contain the bacteria. This phenomenon, coupled with the surge of proinflammatory cytokines eventually led to extensive tissue necrosis with a high bacterial load (Lazar Adler et al., 2009). Therefore, *B. pseudomallei* infected-BALB/c mice highly resemble acute human melioidosis and it is a relatively suitable animal model to study the efficacy of a vaccine against acute *Burkholderia* infection (Macdonald and Speert, 2007).

In contrast to the BALB/c mice, the C57BL/6 exhibit lower secretion of proinflammatory cytokines levels which peaks at a later time (approximately 48–72 h). Higher rate of neutrophils and macrophages infiltration to the infection site have been observed to control the *B. pseudomallei* efficiently to prevent unrestricted spread of the bacteria in C57BL/6 (Lazar Adler et al., 2009). Lower bacterial loads were recovered from organs of infected C57BL/6 mice. These might indicate that C57BL/6 is more resistant to *B. pseudomallei* infection compared to BALB/c mice and as such, is suitable to be used as a chronic model for *B. pseudomallei* infection studies (Macdonald and Speert, 2007).

Long-term latency and recrudescence is one of the important features in human melioidosis which is not presented by BALB/c and C57BL/6 mice upon *B. pseudomallei* infection. Therefore, there is still no appropriate animal model in the study of *B. pseudomallei* latency in host. According to Titball et al. (2008), the TO outbred mice are highly resistant to *B. pseudomallei* infection and they have suggested that the TO outbred mice might be an important model to dissect the mechanisms of long-term latency of *B. pseudomallei* in the host. However, the use of this mouse model has not been widely adopted.

Thus, to date there is no definite suitable rodent models for research in vaccine development especially for human melioidosis. Since melioidosis demonstrates highly varied symptomatic outcome, none of the rodent models available are able to present all stages (i.e., acute, chronic, and latency states) of human melioidosis. Therefore, the lack of suitable testing models pose the major limitation in the study of the effectiveness of vaccine models against *Burkholderia* infections.

## Current strategies and development of *Burkholderia* vaccines

Available in the literature are substantial report on development of vaccines for *B. pseudomallei* but far less for *B. mallei* and *B. cepacia*. It has been indicated that some of the vaccines that have been developed for melioidosis do offer cross-protection to *B. mallei* and *B. cepacia* infections (Sarkar-Tyson et al., 2009).

### Subunit vaccines

Subunit vaccine took advantage of the ability of many different virulence factors of a pathogen to evoke immune responses in order to provide protection to the pathogen. However, preparation of these subunits as a vaccine require large-scale cultures of the pathogenic organism which can be hazardous due to production of aerosols and presence of large amounts of live bacteria and therefore, require extremely tight stringent safety procedures during production. This therefore led to the development of recombinant subunits as alternatives that could be delivered as purified subunit immunogens or DNA encoding the immunogens (Liljeqvist and Stahl, 1999).

#### Burkholderia pseudomallei

Several of the recombinant subunit candidates have been identified to protect against *B. pseudomallei* infection. The most notable candidate being LolC, an outer membrane protein (OMP) of *B. pseudomallei* associated with ATP-binding cassette system (Yakushi et al., 2000). Harland et al. (2007) demonstrated that BALB/c mice immunized with recombinant LolC protein exhibited survivability rate of 80% after six weeks post-infection. Similarly, recombinant LolC protein administered with adjuvant also provided significant protection against challenge using a heterologous strain of *B. pseudomallei* containing the LolC gene. The immunization using LolC with immune-stimulating complex and CpG oligodeoxynucleotide (ODN) stimulated Th-1 type immune response. In the same study, recombinant PotF protein protected 50% of the challenged mice. Immunization with both proteins elicits a Th-1 type immune response in mice which is important in cell-mediated immune response. However, it is not clear whether sterile immunity was achieved in the surviving mice (Table 1).

**Table 1:** **Summary of subunit vaccine development against *B. pseudomallei***.

**References**	**Subunits**	**Immunization**		**Challenge**				**Percentage survived**
		**Dosage and duration**	**Route**	**Animal model**	**Strain**	**Route**	**Dosage**	
Nelson et al. (2004)	Capsular polysaccharide	3 doses in 28 days	Intraperitoneal	BALB/c	NCTC 4845	Intraperitoneal	2 × 10^5^	0% at day 28
	Lipopolysaccharide	3 doses in 28 days	Intraperitoneal	BALB/c	NCTC 4845	Intraperitoneal	2 × 10^5^	50% at day 35
Harland et al. (2007)	ATP binding cassette protein, LolC	3 doses in 28 days	Intraperitoneal	BALB/c	K96243	Intraperitoneal	4 × 10^4^	80% at day 42
	Periplasmic binding protein, PotF	3 doses in 28 days	Intraperitoneal	BALB/c	K96243	Intraperitoneal	4 × 10^4^	50% at day 42
	Oligopeptide binding protein, OppA	3 doses in 28 days	Intraperitoneal	BALB/c	K96243	Intraperitoneal	4 × 10^4^	22% at day 42
Legutki et al. (2007)	Peptide mimotopes of exopolysaccharide	2 doses in 14 days	Intravenous	BALB/c	NCTC 4845	Intraperitoneal	4.7 × 10^4^	0% at day 30
Druar et al. (2008)	BipB, BipC, and BipD proteins	Single dose	Intraperitoneal	BALB/c	Ashdown	Intraperitoneal	970	0% at day 5
Hara et al. (2009)	Outer membrane proteins Omp3 and Omp7	3 doses in 9 weeks	Intraperitoneal	BALB/c	D286	Intraperitoneal	1 × 10^6^	50% at day 21
Su et al. (2010)	Outer membrane protein Omp85	3 doses in 42 days	Intraperitoneal	BALB/c	D286	Intraperitoneal	1 × 10^6^	70% at day 15
Ngugi et al. (2010)	*B. thailandensis* lipopolysaccharide	3 doses in 28 days	Intraperitoneal	BALB/c	K96243	Intraperitoneal	2 × 10^4^	50% at day 35
Burtnick et al. (2011)	Integral surface associated component of type VI secretion system, Hcp1	3 doses in 14 days	Intraperitoneal	Syrian Hamster	K96243	Intraperitoneal	5 × 10^4^	50% at day 42
	Integral surface associated component of type VI secretion system, Hcp2	3 doses in 14 days	Intraperitoneal	Syrian Hamster	K96243	Intraperitoneal	5 × 10^4^	80% at day 42
	Integral surface associated component of type VI secretion system, Hcp3	3 doses in 14 days	Intraperitoneal	Syrian Hamster	K96243	Intraperitoneal	5 × 10^4^	50% at day 42
	Integral surface associated component of type VI secretion system, Hcp4	3 doses in 14 days	Intraperitoneal	Syrian Hamster	K96243	Intraperitoneal	5 × 10^4^	33% at day 42
	Integral surface associated component of type VI secretion system, Hcp5	3 doses in 14 days	Intraperitoneal	Syrian Hamster	K96243	Intraperitoneal	5 × 10^4^	0% at day 42
	Integral surface associated component of type VI secretion system, Hcp6	3 doses in 14 days	Intraperitoneal	Syrian Hamster	K96243	Intraperitoneal	5 × 10^4^	50% at day 42
Nieves et al. (2011b)	Outer membrane vesicle	3 doses in 42 days	Intranasal	BALB/c	1026b	Aerosol	1 × 10^3^	20% at day 14
			Subcutaneous	BALB/c	1026b	Aerosol	1 × 10^3^	60% at day 14

Burtnick et al. (2011) identified another subunit vaccine candidate, Hcp proteins (components of the newly discovered Type 6 Secretion System), which is able to achieve a similar survivability rate as the recombinant LolC protein in the BALB/c mouse model. Six recombinant Hcp proteins (Hcp1–Hcp6) were tested for their ability to confer protection against intraperitoneal *B. pseudomallei* challenge and vaccination using the recombinant Hcp2 protein was found to exhibit a survival rate of 80% at 42 days post-infection (Burtnick et al., 2011). However, vaccination using Hcp1, Hcp3, and Hcp6 only protected 50% of the challenged mice after 42 days of infection while none of the mice immunized with Hcp4 and Hcp5 survived the challenge. It is noteworthy to mention that no bacteria were found in the spleen of the survivors immunized with Hcp1 and Hcp6 while bacteria were recovered from Hcp2-immunized survivors suggesting that sterilizing immunity is possible (Table 1).

Other virulence factors of *B. pseudomallei* such as the surface polysaccharides have also been investigated as subunit vaccines especially two of the most well characterized *B. pseudomallei* surface polysaccharides i.e., the capsular polysaccharide (type I O-PS) and lipopolysaccharide (LPS) (type II O-PS) (Deshazer et al., 1998; Reckseidler et al., 2001; Nelson et al., 2004). Passive immunizations with antibodies to the LPS or capsular polysaccharide were demonstrated to reduce lethality of infection in mice and diabetic rats suggesting that these polysaccharides are of immunological importance (Bryan et al., 1994; Jones et al., 2002). Nelson et al. (2004) suggested the LPS or capsular polysaccharide as potential vaccine candidates for *B. pseudomallei* as vaccination of the BALB/c mice with these polysaccharides followed by an intraperitoneal route of challenge increased the mean time to death (MTTD) compared to the unvaccinated control which exhibited a MTTD of 2.6 days, with 100% mortality occurring by day 11. Lipolysaccharide provided the highest level of protection with a MTTD of 17.6 days and 50% survival at day 35 followed by mice immunized with capsular polysaccharide with a MTTD of 10.5 days and 100% mortality by day 28. Contrastingly, when the mice were challenged using an aerosol route, a slight increase of MTTD was observed as compared with the unvaccinated controls. Additionally, passive transfer of antibody from the immunized mice into the naïve mice also provided protection against subsequent challenges using *B. pseudomallei*.

In another study, since the LPS O-antigen of *B. thailandensis* and *B. pseudomallei* share a high similarity of >90%, the LPS O-antigen of *B. thailandensis* was used as a potential vaccine candidate. Ngugi et al. (2010) demonstrated that vaccination using both *B. thailandensis* LPS and *B. pseudomallei* LPS induced comparable levels of partial of protection against *B. pseudomallei* infections. Vaccination with *B. thailandensis* LPS exhibited 50% survival of the mice at day 35 post-challenge with a MTTD of 32.8 days whereas vaccination using *B. pseudomallei* LPS exhibited 66% survival of mice with a MTTD of 31.5 days. The ability of using the LPS of *B. thailandensis*, the avirulent counterpart of *B. pseudomallei* to provide comparable levels of partial protection against melioidosis is applauded since extraction of LPS from *B. thailandensis* which is a BSL-2 pathogen pose lower costs and more importantly reduced safety risk compared to the extraction from *B. pseudomallei*.

Other subunit vaccine candidates comprising recombinant flagellin antigens, recombinant OMP, outer membrane vesicles (OMV) and peptide mimotypes have also been tested with various degree of protection against *Burkholderia* spp. infection (Chen et al., 2006b; Legutki et al., 2007; Hara et al., 2009; Su et al., 2010; Nieves et al., 2011a). However, no sterile immunity was reported using all these candidates (Table 1). Subunit vaccines should still be considered as potential vaccine candidates as many immunogenic virulence factors of *Burkholderia* species have not yet been evaluated for their protective effects (Mariappan et al., 2010; Vellasamy et al., 2011).

#### Burkholderia mallei

A recent study by Whitlock et al. (2011) achieved 37.5–87.5% survival rate upon challenge by *B. mallei* when mice are immunized with recombinant antigen proteins. These protective antigen proteins were identified by expression library immunization. This method might serves as a model for mass screening of potential vaccine candidate.

#### Burkholderia cepacia

Virulence factors such as proteases, LPS core antigen, flagella antigens, exopolysaccharides, and elastase have been considered as targets for development of vaccine against *B. cepacia* infections. However, none of these antigens are thought to be protective (Doring and Hoiby, 1983; Fujita et al., 1990; Nelson et al., 1993). Recently, a carbohydrate-based potential vaccine has been described which is a trisaccharide based on the repeating unit of a polysaccharide in the LPS of a clinical isolate of *B. cepacia.* However, coupling of trisaccharide or unprotected trisaccharide with a T cell epitope has yet to be developed (Faure et al., 2007).

Using a murine model of chronic pulmonary infection with *B. cepacia*, Bertot et al. (2007) demonstrated that intranasal immunization with OMP can induce specific mucosal immune responses in the respiratory tract, which in turn enhances the clearance of *B. cepacia* and minimize lung inflammatory damage after bacterial challenge. In addition, they have also suggested that the OMP may provide cross-protection against other Bcc members. Immunoglobulin G antibodies to *B. cepacia* OMP have been detected in sera of CF patients colonized with both *B. cepacia* and *P. aeruginosa*, suggesting cross-reactivity between the OMPs of these organisms (Aronoff et al., 1991).

In addition, Makidon et al. (2010) confirmed the findings of Bertot et al. (2007) and demonstrated significant response to the 17 kDa OmpA lipoprotein. In their study, it was found that immunized mice were protected against pulmonary colonization with *B. cepacia* and they have also identified a new immunodominant epitope, a 17 kDa OmpA-like protein which is highly conserved between *Burkholderia* and *Ralstonia* spp. In addition, serum analysis showed robust IgG and mucosal secretory IgA immune responses in vaccinated versus control mice and this suggest that the antibodies had cross-neutralizing activity against Bcc. Makidon et al. (2010) have also suggested that the 17 kDa OmpA-like protein shows potential for future vaccine development and provides a rational basis for vaccines using recombinant OMP mixed with nano-emulsion as an adjuvant.

More recently, 18 immunogenic proteins from *B. cepacia* culture supernatant that reacted with mice antibodies raised to *B. cepacia* inactivated whole bacteria, OMP, and culture filtrate antigen were suggested to be potential molecules as a diagnostic marker or putative candidates for development of vaccine and therapeutic intervention against *B. cepacia* infections (Mariappan et al., 2010). Additionally, secretory proteins of *B. cepacia* have been suggested as possible targets for the development of new strategies to control *B. cepacia* infection using agents that can block their release (Mariappan et al., 2011).

Metalloproteases are also being considered as potential effective candidates for vaccine development (Corbett et al., 2003). Furthermore, it was demonstrated that immunizations of mice using a conserved zinc metalloprotease peptide decreased the severity of *B. cepacia* infection and the lung damage was reduced by 50% on challenge with a *B. cepacia* strain after immunization with this peptide (Corbett et al., 2003). Additionally, BLAST analysis demonstrated that metalloprotease shared a 96% and above homology with the metalloprotease of *B. pseudomallei* K96243. Therefore, this protein has been suggested to be used to cover immunization for both *B. cepacia* and *B. pseudomallei* to cross-protect (Mariappan et al., 2010).

### Live attenuated vaccines

Live vaccines have been widely used for vaccination against infectious diseases, such as tuberculosis, cholera, mumps, and measles. Previously, live attenuated vaccines were developed from attenuated strains which have undergone passages in laboratory conditions or immunologically related species/microorganism that does not use human as the target host. Live attenuated vaccines are able to replicate and are recognized as foreign by human body without causing any pathological or lethal effects. Empirical approaches used for live vaccines development are time-consuming and the basis of attenuation is usually undefined. The advancement of modern DNA recombinant technology enables researchers to specifically inactivate essential gene(s) involved in pathogenesis, which leads to the development of well-defined attenuated live vaccines (Clare and Dougan, 2004). Live attenuated vaccines are considered the most ideal vaccine with great advantage due to the ability to elicit wide range of immune responses. *Burkholderia* pathogens are cytosolic intracellular bacteria whereby the antigenic proteins present in the cytosol after endosomal escape will be processed by the proteasome in the endogenous pathway and presented to CD8^+^ T cells by class I major histocompatibility complex (MHC). In contrast, inactivated vaccines are generally unable to induce CD8^+^ cytotoxic T cell immunity, which is essential in clearing intracellular pathogens (Seder and Hill, 2000). Live attenuated vaccines require lesser or even only a dose of vaccination for long lasting immune protection and without the use of adjuvant.

#### Burkholderia pseudomallei

To date, genes involving capsular polysaccharide synthesis and amino acid synthesis pathways (for example, shikimic pathway, and branch chain amino acids synthesis) are the main targets for creating attenuated variant. Acapsular mutant of *B. pseudomallei* (strain 1E10) created by Atkins et al. (2002a) showed no immunization protective effect toward BALB/c mice challenged by *B. pseudomallei* strain 576. The *gmhA* and *wcbJ* mutant strains which are defective in capsular polysaccharide synthesis were used to immunize BALB/c mice. The immunized mice were partially protected from *B. pseudomallei* challenge; however, sterile immunity was not achieved.

Several other genes involved in essential amino synthesis pathway have also been knocked-out and exhibited attenuated virulence. The *B. pseudomallei* strain 2D2 with defective acetolactate synthase (*ilvI*) gene identified from a mutant library are a branch amino acid auxotroph. Mice vaccinated with 2D2 survived longer compared to naïve mice when challenged with *B. pseudomallei* 576 and BRI but not protected toward *Francisella tularensis* challenge (Atkins et al., 2002b). In another study performed by Haque and his team (2006) using *B. pseudomallei* 2D2 as the vaccine model have demonstrated that vaccinated mice elicited CD4^+^ immune reaction as a protective mechanism against *B. pseudomallei* infections. With the same vaccination model, intranasal route of vaccination was shown to confer better protection toward pulmonary *B. pseudomallei* infection which was associated with higher production of interferon-γ (IFNγ) compared to intraperitoneal administration (Easton et al., 2011). The same study also demonstrated that vaccination incorporated with intranasal CpG ODN treatment extended survival ability of vaccinated mice, highly reduced lung bacterial load and delayed onset of bacterial sepsis after challenge. Similarly, *ilvI* gene, phosphoserine aminotransferase (*serC*) (Rodrigues et al., 2006), dehydroquinate synthase (*aroB*) (Cuccui et al., 2007), chorismate synthase (*aroC*) (Srilunchang et al., 2009), and aspartate semialdehyde dehydrogenase (*asd*) (Norris et al., 2011a) genes have also been utilized as gene targets to develop amino acid auxotrophs for vaccination studies in melioidosis (Table 2).

**Table 2:** **Summary of live attenuated vaccine development against *B. pseudomallei***.

**References**	**Mutated gene**	**Immunization**			**Challenge**				**Percentage survived**
		**Dosage**	**Duration**	**Route**	**Animal model**	**Strain**	**Dosage**	**Route**	
Atkins et al. (2002a)	Mannosyltransferase (1E10 strain)	10^6^	5 weeks	Intraperitoneal	BALB/c	576	10^4^	Intravenous	0% at day 18
Atkins et al. (2002b)	Acetolactate synthase (*ivlI*) (2D2 strain)	10^6^	5 weeks	Intraperitoneal	BALB/c	576	10^6^	Intraperitoneal	80% at day 35
Stevens et al. (2004)	Type III secretion system cluster 3 translocator protein (*bipD*)	10^4^	5 weeks	Intraperitoneal	BALB/c	576	10^4^	Intraperitoneal	60% at day 70
Ulett et al. (2005)	Wild type (CL04 strain)	8.5 × 10^3^	2 weeks	Unknown	BALB/c	NCTC 13178	7.2 × 10^2^	Unknown	40% at day 14
			Immunization: 1 week Booster: 1 week						100% at day 14
Haque et al. (2006)	Acetolactate synthase (*ivlI*) (2D2 strain)	10^6^	5 weeks	Intraperitoneal	BALB/c	576	10^6^	Intraperitoneal	40% at day 75
Rodrigues et al. (2006)	Phosphoserine aminotransferase (*serC*) (4D6 strain)	10^5^	5 weeks	Intraperitoneal	BALB/c	K96243 and 576	10^4^	Intraperitoneal	80% at day 25
Barnes and Ketheesan (2007)	Wild type (NTCC 13179)	290	Immunization: 10 days First booster: 20 days Second booster: 15 days	Subcutaneous	BALB/c	NTCC 13178	20	Intravenous	81.80% at day 40
Cuccui et al. (2007)	Dehydroquinate synthase (*aroB*) (13B11 strain)	10^5^	5 weeks	Intranasal	BALB/c	K96243	10^3^	Intranasal	0% at day 8
		10^6^							0% at day 8
Breitbach et al. (2008)	*purN*	10^5^	3 weeks	Intraperitoneal	BALB/c	E8	10^5^	Intraperitoneal	100% at day 30
	*purM*								100% at day 28
Srilunchang et al. (2009)	Chorismate synthase (*aroC*)	3 × 10^7^	Immunization: 1 week Booster: 1 week	Intraperitoneal	BALB/c	A2	5 × 10^2^	Intraperitoneal	0
							5 × 10^3^		0
							5 × 10^4^		0
		5 × 10^8^			C57BL/6		6 × 10^3^		80% at day 150
							6 × 10^4^		60% at day 150
							6 × 10^5^		20% at day 150
Norris et al. (2011b)	Aspartate semialdehyde dehydrogenase (*asd*)	10^7^	Immunization: 3 weeks Booster: 2 weeks	Intranasal	BALB/c	1026b	4 × 10^3^	Intranasal	20% at day 50
Easton et al. (2011)	Acetolactate synthase (*ivlI*) (2D2 strain) combining with CpG	10^5^	5 weeks	Intranasal	BALB/c	576	3 × 10^2^	Intranasal	unknown
Cuccui et al. (2012)	Sedaheptulose-7-phosphate isomerase (*gmhA*)	10^3^	5 weeks	Intranasal	BALB/c	K96243	10^3^	Intranasal	0% at day 35
	Reductase (*wcbJ*)								33% at day 35

The type 3 secretion system cluster 3 (TTSS3) (Stevens et al., 2004) encodes important virulence factors which are highly involved in *B. pseudomallei* pathogenesis (Lazar Adler et al., 2009). Based on this, a mutant defect in the translocator protein gene, *bipD*, was constructed by Stevens et al. (2004) for BALB/c mice immunization and the immunized mice exhibited partial protection against *B. pseudomallei* infections. Besides the genes involved in amino acid synthesis, polysaccharide synthesis, and T3SS, genes involved in purine synthesis pathway are also candidates for construction of live attenuated vaccines. The *purN* defective mutant strains were used for immunization in the study performed by Breitbach et al. (2008). Protection against *B. pseudomallei* challenge was demonstrated in immunized BALB/c mice (Breitbach et al., 2008) (Table 2). Another mutant strain created in the same study (*purM*-knock out strain) demonstrated dosage-dependent protection whereby mice immunized with higher dosage of *purM* mutant exhibited better protection than that of lower dosage (Table 2).

Interestingly, *B. pseudomallei* strain CL04 (isolated from a chronic melioidosis patient) (Ulett et al., 2005) and NTCC 13179 (Barnes and Ketheesan, 2007) exhibited natural attenuated virulence and protection properties when immunized BALB/c mice were challenged with virulent *B. pseudomallei* strains (Table 2). However, CL04 and NTCC 13179 are wild type *B. pseudomallei* strains isolated from melioidosis patients and these strains are not suitable to be used as the live attenuated vaccine candidates.

#### Burkholderia mallei

Similar to *B. pseudomallei*, the mutant strain 1E10, an acapsular mutant of *B. mallei* (strain DD3008) exhibited no vaccination protection toward challenge against *B. mallei* ATCC 23344 (Deshazer et al., 2001). This might be due to the development of IgG_1_ (Th2-like immunoglobulin) antibody response in vaccinated mice (Ulrich et al., 2005). *B. mallei* knockout strain of *ilvI* (strain ILV1) was also constructed in order to develop vaccines against *B. mallei* and to study the effect of vaccination on glanders. As reported by Ulrich and others (2005), *B. mallei* ILV1-vaccinated mice developed IgG_2_ (Th1-like immunoglobulin) antibody response which conferred resistance toward *B. mallei* infection. However, ILV1 vaccination does not confer sterile immunity and the vaccinated mice developed splenomegaly following challenge with live bacteria (Table 3). Apart from the *ilvI* gene, gene knockouts for capsule production (*wcbB*) and carboxyl-terminal protease-encoded gene (*ctpA*) also demonstrated attenuated virulence of *B. mallei*. Administration of the *wcbB* and *ctpA* knockout strains conferred protection against wild type *B. mallei* in vaccinated animal models (Table 3).

**Table 3:** **Summary of live attenuated vaccine development against *B. mallei***.

**References**	**Mutated gene**	**Immunization**			**Challenge**				**Percentage survived**
		**Dosage**	**Duration**	**Route**	**Animal model**	**Strain**	**Dosage**	**Route**	
Deshazer et al. (2001)	*wcb*	33285-766667	3 weeks	Aerosol	BALB/c	ATCC 23344	Approx. 1.8 × 10^8^	Aerosol	20% at day 21
Ulrich et al. (2005)	Acetolactate synthase (*ilvI*)	4.7–7.3 × 10^4^	Immunization: 3 weeks Booster: 3 weeks	Aerosol	BALB/c	ATCC 23344	5 × 10^3^	Aerosol	25% at day 30
							4.4 × 10^5^		50% at day 30
Bandara et al. (2008)	Carboxyl-terminal protease (*ctpA*)	4.4 × 10^5^	5 weeks	Intraperitoneal	CD1	ATCC 23344	6.6 × 10^5^	Intraperitoneal	75% at day 15

Despite accumulating data on live attenuated vaccines against *B. pseudomallei* and *B. mallei*, the analysis is rather complicated due to the different immunization protocols, wild type challenge dosages, route of administration, and animal model used. Therefore, the results are not comparable in order to determine the most suitable live attenuated vaccine candidate for use as potential *Burkholderia* vaccines (Sarkar-Tyson and Titball, 2010).

### Killed whole cell vaccines

Killed whole cell vaccines are non-living pathogens which remain immunogenic for host immune system to recognize as “foreign.” These vaccines have been widely used for the prevention of anthrax, Q fever, and whooping cough (Ada, 2001). This type of vaccine is known to induce a narrow range of immune responses due to the inability to replicate in the host. On the other hand, the inability to replicate is also a major advantage for the killed whole cell vaccines; making them an extremely safe prophylactic stimulation of the immune system and as such suitable for vaccinations on immunocompromised individuals. These vaccines are also good candidates for inducing humoral immunity to produce neutralizing antibodies. It is generally known that killed whole cell vaccines do not stimulate strong cellular-mediated immunity due to the fact that the antigens are not processed by an endogenous pathway and presented to the T cells by class I MHC. Hence, multiple doses of vaccination and adjuvants are required for long lasting protection (Ada, 2003). Experimental testing of the potency of kill whole cell vaccines in *Burkholderia* spp. has been investigated employing various animal models, administration and challenge routes, inactivation protocols, and adjuvants.

#### B. pseudomallei

Inactivated *Burkholderia* pathogens confer different degrees of protections toward *B. pseudomallei* and *B. mallei* infections. *B. pseudomallei, B. mallei*, and *B. thailandensis* are phylogenetically closely related and hence, cross protections are highly possible. Study conducted by Sarkar-Tyson et al. (2009) has demonstrated that immunization by heat-inactivated *B. mallei* and *B. thailandensis* conferred protection toward *B. pseudomallei* infections. In fact, BALB/c mice immunized by heat-inactivated *B. mallei* ATCC 23344 exhibited better protection (i.e., higher survival percentage and longer MTTD) against *B. pseudomallei* strain K96243 infection compared to mice immunized by heat-killed *B. pseudomallei* strain K96243 and 576. Barnes and Ketheesan (2007) also, demonstrated that heat-killed *B. pseudomallei* immunization via subcutaneous route does not protect immunized mice from subsequent challenge but heat-killed *B. pseudomallei* co-injected with culture filtrate antigen significantly increase the protection toward subsequent challenge (Table 4).

**Table 4:** **Summary of killed whole cell vaccine development against *B. pseudomallei***.

**References**	**Bacteria**	**Strain**	**Inactivation mode**	**Immunization**				**Animal model**	**Challenge**			**Percentage survival**
				**Dosage**	**Duration**	**Adjuvant/Additives**	**Route**		**Strain**	**Dosage**	**Route**	
Healey et al. (2005)	*B. pseudomallei*	NCTC 4845	Heat	5.3 × 10^4^	Immunization: 4 weeks Booster: Unknown	DC	Intradermal	BALB/c	NCTC 4845	5 × 10^3^	Intranasal	60% at day 35
				5 × 10^3^		Monophosphoryl lipid A-trehalose dicorynomycolate						
Elvin et al. (2006)	*B. pseudomallei*	K96243	Heat	10^4^	Immunization: 3 weeks Booster: 2 weeks	DC	Intradermal	BALB/c	K96243, NCTC 4845, and 576	10^4^	Intraperitoneal	20% (K96243), 10% (NCTC 4845), 20% (576) at day 42
						DC, CpG 1826 pulse together with immunogen						90% (K96243), 70% (NCTC 4845), 70% (576) at day 42
						DC, CpG 1826 as adjuvant						70% (K96243), 60% (NCTC 4845), 60% (576) at day 42
Barnes and Ketheesan (2007)	*B. pseudomallei*	NCTC 13179	Heat	290	Immunization: 10 days First booster: 20 days Second booster: 15 days	None	Subcutaneous	BALB/c	NCTC 13178	20	Intravenous	0% at day 40
			Heat	90		5 μg/4 μL culture filtrate antigens						66.7% at day 40
Sarkar-Tyson et al. (2007)	*B. pseudomallei*	K96243	Heat	10^8^	Immunization: 2 weeks First booster: 2 weeks Second booster: 5 weeks	None	Intraperitoneal	BALB/c	K9243	6.05 × 10^4^	Intraperitoneal	40% at day 35
		K96243 ΔwbiA::kan										80% at day 35
		K96243 ΔwcbH::kan										70% at day 35
		K96243 ΔBPSS0421:: kan										50% at day 35
		K96243 ΔBPSS1833:: kan										50% at day 35
Sarkar-Tyson et al. (2009)	*B. pseudomallei*	576	Heat	10^7^	Immunization: 2 weeks First booster: 2 weeks Second booster: 5 weeks	None	Intraperitoneal	BALB/c	K96243	1.4 × 10^4^	Intraperitoneal	50% at day 44
	*B. pseudomallei*	K96243										65% at day 44
	*B. mallei*	ATCC 23344										70% at day 44
	*B. thailandensis*	E27										60% at day 44
Sarkar-Tyson et al. (2009)	*B. pseudomallei*	576	Heat	10^7^	Immunization: 2 weeks First booster: 2 weeks Second booster: 5 weeks	None	Intraperitoneal	BALB/c	K96243	92	Aerosol	Not specified (1)
	*B. pseudomallei*	K96243										Not specified (2)
	*B. mallei*	ATCC 23344										Not specified (3)
	*B. thailandensis*	E27										Not specified (4)
Henderson et al. (2011)	*B. pseudomallei*	1026b	Heat	10^5^	Immunization: 10 days Booster: 14 days	None	Intranasal	BALB/c	1026b	7500	Intranasal	44% at day 40
						Cationic liposome complexed with non-coding plasmid DNA						100% at day 40

DC, dendritic cell.

(1) MTTD, 9.75 days; (2) MTTD, 8 days; (3) MTTD, 17.2 days; (4) MTTD = 7.4 days.

Other studies have demonstrated that the presence of capsular polysaccharide and LPS were able to affect the immunogenicity of heat-inactivated *B. pseudomallei* (Sarkar-Tyson et al., 2007). Heat-killed capsular polysaccharide mutant (*wcbH* knockout) and LPS O antigen mutants (*wbiA* knockout) demonstrated higher vaccination protection against *B. pseudomallei* (70% and 80% after day 35, respectively) in comparison to mice vaccinated with heat-killed putative type III O-polysaccharide mutant (*BPSS0421* knockout) and putative type IV O-polysaccharide mutants (*BPSS1833* knockout) in the study of Sarkar-Tyson and collaborators (2007) (Table 4). The authors have postulated that depletion of capsular polysaccharide and LPS might leads to the exposure of hidden immunogens or upregulation of the less abundant surface polysaccharides. The use of cationic liposomes complexed with non-coding plasmid DNA as mucosal adjuvant with heat-killed *B. pseudomallei* has also been found to increase the survival rate of challenged BALB/c mice to 100% (after day 40) compared to BALB/c only immunized with heat-killed *B. pseudomallei* (Henderson et al., 2011) (Table 4).

Dendritic cells are strong professional antigen presenting cells (APC) which induce strong adaptive immunity, especially T cells-based immunity. Therefore, delivering antigen-primed dendritic cells into human is a potential method to induce vaccinated protection. Thus, pulsing of inactivated *B. pseudomallei* into dendritic cells was performed and reported. Mice immunized by heat-killed *B. pseudomallei* pulsed dendritic cells demonstrated significant stronger cell-mediated immune responses compared to mice immunized by heat-killed *B. pseudomallei*. The addition of CpG ODN with heat-killed *B. pseudomallei* during priming of dendritic cells or as adjuvant demonstrated increase in the secretion of IFNγ (indicating the stimulation of Th1 immune responses) and anti-*B. pseudomallei* antibody production (Healey et al., 2005; Elvin et al., 2006) (Table 4).

#### Burkholderia mallei

Amemiya et al. (2002), demonstrated that BALB/c mice immunized by heat-inactivated *B. mallei* strain ATCC 23344 with Alhydrogel as adjuvant showed mixed Th1- and Th2-like cytokine responses in spleenocytes and Th2-like IgG responses. However, these vaccinated mice were not protected from the *B. mallei* challenge. This study eventually led to the investigation of the protective effects conferred by co-administration of interleukin (IL) 12 with heat-killed *B. mallei* and Alhydrogel adjuvant. Incorporation of IL12 increased the secretion of IFNγ and IL10 while at the same time increasing the production of anti-*B. mallei* IgG2. These collectively indicate that the Th1 response was stimulated in BALB/c mice challenged with live *B. mallei*. Th1 immune response elicited after inclusion of IL12 in the vaccination regime has conferred partial protection against *B. mallei* challenged via intraperitoneal route (Amemiya et al., 2006). However, studies reported by Whitlock and others (2008) demonstrated that sterilizing immunity was not achieved in immunized BALB/c. Interestingly, the greatest protection against *B. mallei* was not conferred by heat-inactivated bacteria from the same species but from a closely related species as demonstrated by Sarkar-Tyson et al. (2009). They observed higher resistance exhibited by BALB/c mice immunized with heat-inactivated *B. pseudomallei* K96243 compared to the BALB/c mice immunized with heat-inactivated *B. mallei* ATCC 23344 when challenged with *B. mallei* ATCC 23344 (Table 5).

**Table 5:** **Summary of killed whole cell vaccine development against *B. mallei***.

**References**	**Bacteria**	**Strain**	**Inactivation mode**	**Immunization**				**Animal model**	**Challenge**			**Percentage survival**
				**Dosage**	**Duration**	**Adjuvant/Additives**	**Route**		**Strain**	**Dosage**	**Route**	
Amemiya et al. (2002)	*B. mallei*	GB15.1-2 (1)	Heat	2.6 × 10^7^	Immunization: 3 weeks Booster: 4 weeks	100 μg Alhydrogel	Subcutaneous	BALB/c	GB15.1-2 (1)	2.3 × 10^8^	Intraperitoneal	0% at day 21
			UV									25% at day 21
										2.8 × 10^8^		20% at day 21
									CapGB15 (2)	2.8 × 10^8^		100% at day 21
										6.5 × 10^8^		80% at day 21
Amemiya et al. (2006)	*B. mallei*	ATCC 23344	Heat	1.3 × 10^7^	Immunization: 3 weeks Booster: 3 weeks	100 μg Alhydrogel and IL12	Subcutaneous	BALB/c	GB15.1-2 (1)	10^8^	Intraperitoneal	20–60% at day 21
Whitlock et al. (2008)	*B. mallei*	ATCC 23344	Heat	10^5^	2 weeks	None	Intraperitoneal	BALB/c	ATCC 23344	2 × 10^7^	Intraperitoneal	40% at day 12
Sarkar-Tyson et al. (2009)	*B. pseudomallei*	576	Heat	10^7^	Immunization: 2 weeks First booster: 2 weeks Second booster: 5 weeks	None	Intraperitoneal	BALB/c	ATCC 23344	6.3 × 10^3^	Aerosol	Not specified (3)
	*B. pseudomallei*	K96243	Heat									Not specified (4)
	*B. mallei*	ATCC 23344	Heat									Not specified (5)
	*B. thailandensis*	E27	Heat									Not specified (6)

(1) B. mallei strain ATCC 23344 serially passaged three times through Syrian hamsters; (2) Capsule-negative mutant of B. mallei strain GB15.1-2; (3) MTTD, 13.78 days; (4) MTTD, 23.1 days; (5) MTTD, 13.7 days; (6) MTTD, 6.3 days.

### DNA vaccines

Immunization using DNA is used to efficiently stimulate humoral and cellular immune responses to protein antigens. When a genetic material is directly injected into the host, a small amount of the host's cells will express the related gene products. This inappropriate gene expression within the host has important immunological consequences, resulting in the specific immune activation of the host against the gene delivered antigen (Koprowski and Weiner, 1998).

Currently the only DNA vaccine developed against any *Burkholderia* spp. utilizes the *fliC* gene of *B. pseudomallei*. Chen et al. (2006b) demonstrated that mice vaccinated with plasmid DNA using mammalian expression vector pcDNA3/fliC gene survived better than mice vaccinated with recombinant FliC protein (83% vs. 50%). Plasmid DNA encoding the flagellin gene was able to generate high levels of specific antibodies in which upregulation of IFN-γ and increase in the IgG2a/IgG1 ratio was observed. However, generally the DNA vaccines were reported to induce weaker immune response in humans (Liu, 2011). It was demonstrated that immunization of mice with priming of immuno-stimulatory CpG motifs and pcDNA3/fliC further enhances the survivability to 93.3% when challenged with *B. pseudomallei* (Chen et al., 2006a).

### Potential vaccine approaches

When BALB/c mice were immunized with heat-inactivated *B. thailandensis* significant delay in the MTTD post-challenged with *B. pseudomallei* compared to non-immunized BALB/c mice were observed. Therefore, *B. thailandensis* may be considered a potential vaccine candidate, following genetic modification in order to express the immunogenic proteins from *B. pseudomallei* and *B. mallei*. In addition, Iliukhin et al. (2004), demonstrated that immunized animal models conferred resistance to *B. pseudomallei* and *B. mallei* following subcutaneous administration of live attenuated *F. tularensis*. It was suggested that *F. tularensis* may also be considered as a potential candidate for *Burkholderia* vaccines development since it is also a cytoplasmic facultative intracellular bacteria, similar to *Burkholderia* pathogens.

To date, development of live attenuated vaccines for *Burkholderia* spp. has only exploited the knockout of one gene at a time using techniques such as genetic alterations of bacteria via transformation, conjugation, or transduction. Therefore, reversion from attenuated strain to wild-type strain can occur in the environment and hence a major concern in the use of live attenuated bacteria as vaccine candidates. Two or more independent site-specific knockout should be considered as demonstrated in *S. typhi* strain CVD 908-*htrA* and *Shigella flexneri* strain CVD 1207. This approach needs to be treated with caution as it may pose the risk of over attenuation leading to a reduced immunogenicity of the attenuated vaccine as demonstrated for *S. typhi* strain 541Ty (Clare and Dougan, 2004).

It is well-known that heat and chemical treatment on pathogens for killed whole cell vaccines poses risk of affecting the immunogenicity. An alternative approach to inactivate Gram-negative pathogens like *Burkholderia* spp. without affecting the immunogen epitopes is the creation of a bacterial ghost by employing PhiX174 *E* gene lytic mechanism. The lysis *E* gene encodes a hydrophobic protein which produces transmembrane channels and releases the cytoplasmic content of the pathogen. This leaves the empty bacterial cell envelop which retains the immunogenicity and is able to stimulate APCs for major histocompatibility presentation (Haslberger et al., 2000; Talebkhan et al., 2010). This technique has been exploited in a variety of Gram-negative bacterial vaccine development (Szostak et al., 1996). Since there are a number of lytic *Burkholderia* bacteriophages that have been reported, exploiting the next generation sequencing and bioinformatics techniques, a more sophisticated lysis system could be employed for the creation of bacterial ghost of *Burkholderia* pathogens, as tools for killed whole cell vaccine development.

## Cost effectiveness and efficacy of a *Burkholderia* vaccine

In a recent paper, Peacock et al. (2012) emphasized the importance of cost-effectiveness of vaccines against *B. pseudomallei*. The study demonstrated the need to produce cost savings or at least potential savings depending on the level of efficacy of the vaccine, vaccination coverage rates, the population's risk profile, the cost of the vaccine, and whether future medical costs for additional years of life gained were included in the calculations.

Vaccines intervention for the control of endemic *Burkholderia* spp. should also be targeted where the most reliable estimates of the incidence of *Burkholderia* spp. infections occur. The high-risk groups such as diabetics and those with chronic kidney or lung disease could be considered as primary targets for *Burkholderia* spp. vaccine trials. In addition, occupational risk populations which include farmers, construction workers, and military personnel also need to be considered as target groups. Taking the occupational and economic-based evaluation into consideration, there is a necessity for the vaccine to be used in a low income community since *Burkholderia* spp. infections are also known as the “poor man's disease.”

Among the high-risk populations, in the community of an endemic area, vaccination against *Burkholderia* pathogens can be associated with less frequent hospitalizations and with direct savings in health care costs. Overall, this will reduce the burden of the government in terms of health care cost. Reduction in disease severity alone would be predicted to improve outcome in view of the high mortality rate. However, one disadvantage may be that generating protective immunity in individuals with such underlying diseases may be difficult to achieve since several *Burkholderia* spp. infections spread from person-to-person. Emergency measures should be taken as part of the biodefense efforts by focusing on increasing the awareness amongst the medical community, improving the diagnostic capabilities and preparation of national response protocols. It has been suggested that vaccinating the risk group against *Burkholderia* spp. infections is more cost effective than many other preventive and therapeutic interventions (Peacock et al., 2012).

## Future direction

Despite different studies and strategies being used to identify effective vaccine candidates for *Burkholderia* spp., to date, no reliable and conveniently measured correlates of vaccine-induced efficacy against these bacteria and/or other intracellular pathogens have been identified. Most importantly, researchers have been unable to identify and measure the immune responses that may be used to predict vaccines that will protect against the intracellular pathogens. While some of the vaccine candidates are able to induce substantial humoral response, some lacked the ability to evoke strong cellular immune responses or the ability to protect against infection with the *Burkholderia* spp. Perhaps, better understanding of the specific mechanisms by which members of the *Burkholderia* spp. subvert host defenses and survive intracellularly will provide a stronger platform for identification of an effective vaccine candidate. Another limiting factor is the difficulties in generating protective immunity in immunocompromised individuals with underlying diseases such as diabetes and chronic lung disease may be difficult to achieve. Such correlation would extensively aid in the design of new vaccines for Gram-negative intracellular pathogens in general and *Burkholderia* spp. in specific.

### Conflict of interest statement

The authors declare that the research was conducted in the absence of any commercial or financial relationships that could be construed as a potential conflict of interest.
